# Integrating Muscle Health in Predicting the Risk of Asymptomatic Vertebral Fracture in Older Adults

**DOI:** 10.3390/jcm10051129

**Published:** 2021-03-08

**Authors:** Yu-Ching Lin, Yu-Hsiang Juan, Wing P. Chan, Kun-Yun Yeh, Alice M. K. Wong, Chen-Ming Sung, Yu-Jr Lin, Shu-Chen Chang, Fang-Ping Chen

**Affiliations:** 1 Department of Medical Imaging and Intervention, Chang Gung Memorial Hospital at Keelung, College of Medicine, Chang Gung University, Keelung 222, Taiwan; yuching1221@gmail.com (Y.-C.L.); gtsung@cgmh.org.tw (C.-M.S.); 2 Department of Medical Imaging and Intervention, Chang Gung Memorial Hospital at Taoyuan, Institute for Radiological Research, Chang Gung University, Taoyuan 333, Taiwan; jonat126@yahoo.com.tw; 3 Department of Radiology, Wan Fang Hospital, Taipei Medical University, Taipei 116, Taiwan; wingchan@tmu.edu.tw; 4 Department of Radiology, School of Medicine, College of Medicine, Taipei Medical University, Taipei 110, Taiwan; 5 Department of Internal Medicine, Division of Hemato-Oncology, Chang Gung Memorial Hospital, College of Medicine, Keelung & Chang Gung University, Keelung 222, Taiwan; yehtyng@gmail.com; 6 Department of Physical Medicine and Rehabilitation, Chang Gung Memorial Hospital at Taoyuan, Taoyuan 333, Taiwan; alicewong.mk@gmail.com; 7 Healthy Aging Research Center, Chang Gung University, Taoyuan 333, Taiwan; 8 Research Services Center for Health Information, Chang Gung University, Taoyuan 333, Taiwan; linyufour@gmail.com (Y.-J.L.); kathelinchang@gmail.com (S.-C.C.); 9 Department of Obstetrics and Gynecology, Chang Gung Memorial Hospital, Keelung and Chang Gung University, Keelung 222, Taiwan; 10 Keelung Osteoporosis Prevention and Treatment Center, Keelung 222, Taiwan

**Keywords:** vertebral fracture, sarcopenia, bone quantity, bone quality, fracture risk

## Abstract

Background: The utility of muscle health for predicting asymptomatic vertebral fracture (VF) is uncertain. We aimed to determine the effects of muscle health on bone quantity and quality in the older adults and to integrate these factors into a predictive model for VF. Methods: We prospectively recruited participants with a body mass index <37 kg/m^2^. The total lean mass (TLM), appendicular skeletal muscle index, presence of sarcopenia, and bone mineral density were determined by dual-energy X-ray absorptiometry, and bone quality by the trabecular bone score (TBS). VF was diagnosed based on spine radiography. Results: A total of 414 females and 186 males were included; 257 participants had VF. Lower TLM was significantly associated with poorer bone quantity and quality in both males and females. A low TBS (OR: 11.302, *p* = 0.028) and sarcopenia (Odds ratio (OR): 2.820, *p* = 0.002) were significant predictors of VF in males, but not bone quantity. Moreover, integrating TBS and sarcopenia into the predictive model improved its performance. Conclusions: Although TLM was associated with bone quantity and quality in both sexes, sarcopenia and a low TBS were significant predictors of asymptomatic VF only in male participants.

## 1. Introduction

Vertebral fracture (VF) is the most common type of osteoporotic fracture [1,2], and its occurrence is both age- and sex-dependent [3]. Osteoporotic VF can have a considerable impact on health-related quality of life, since it is associated with a five-fold increase in the risk of a future VF and a three-fold increase in the risk of a future hip fracture [4,5]. Furthermore, previous studies reported a high prevalence of asymptomatic osteoporotic VF in the older adults, which can reach up to one-fourth in those aged 50 years or more [6,7]. Thus, diagnosing VF solely based on symptoms may be insufficient. There is a need for a more efficient tool to detect asymptomatic osteoporotic VF at an early stage in the older adults, to facilitate early osteoporotic treatment, decrease the risk of future fracture development and improve quality of life [8].

Bone fragility, due to both poor bone quantity and quality, was previously considered the main cause of VF [9,10]. Bone quantity is often determined based on bone mineral density (BMD), while bone quality can be determined by assessment of the trabecular microarchitecture, bone turnover, and cortical macrogeometry [11,12]. Besides bone quantity and quality, there is also increasing emphasis on the relationship between bone fragility and muscle health, where muscle and bone have common genetic, nutritional, and hormonal determinants [13]. Likewise, a previous study reported a significant association between sarcopenia and lumbar spine or total hip BMD [14]. Sarcopenia increases the risk of developing osteoporosis [13] and fracture [15]. In 2009, the term “sarco-osteopenia” was coined to emphasize that both weak bones and weak muscles may contribute to fractures in the older adults [16].

However, the literature discussed above focused mainly on the relationship between muscle health and bone quantity and lacked bone quality assessment. Weakened muscle contraction may decrease longitudinal stress on the bones involved and subsequently decrease the number and quality of bone trabeculae [17]. Thus, a comprehensive assessment of asymptomatic VF risk should include evaluation of bone mineralization, bone quality, and muscle. In addition, there has been limited investigation concerning the potential utility of sarcopenia for predicting asymptomatic VF. Therefore, the aim of this study is two-fold: to understand the impact of muscle health on bone quantity and quality in older adults and to integrate sarcopenia and bone quality and quantity into a predictive model of asymptomatic VF.

## 2. Material and Methods

### 2.1. Subjects

Prospective recruitment was approved by our institutional review board (IRB approval numbers 201800598B0 and 201800841B0). Written informed consent was obtained from all participants. The target population was residents of the North District in Taiwan who participated in a community osteoporosis screening project. The participants had no known history of sarcopenia, osteopenia, osteoporosis, or VF. There were two patients who underwent transpedicular screw fixation for spondylolisthesis, but VF was not visible in images taken during their surgeries. Thus, any VFs diagnosed during this study were either asymptomatic or newly developed. We only enrolled participants who were willing to undergo clinical and imaging studies of bone fragility, VF, and muscle health. Bone fragility evaluations included BMD for determination of bone quantity and the trabecular bone score (TBS) for determination of bone quality. VF was evaluated on lateral thoracolumbar spine radiographs. Muscle health evaluations included body mass index (BMI), total body composition (TBC), and handgrip strength (HS). All imaging evaluations were performed on the same day.

A total of 615 participants were recruited; we excluded those aged < 60 years (*n* = 13) and those with a BMI > 37 kg/m^2^ (*n* = 2). This was because we were only interested in older adults in this study, and TBS analysis is not considered valid for those with a BMI > 37 kg/m^2^ [18]. Demographic data, including age, sex, body weight, height, and BMI were collected. Moreover, history of falls was recorded, defined as two or more falls in the past year.

### 2.2. Anthropometric Measurements

Weight, height, and BMI measurements were obtained with the participants clad in light clothing and barefooted. BMI was defined as the weight in kilograms divided by the height in meters squared (kg/m^2^).

### 2.3. Bone Fragility Evaluation

#### Imaging Protocols

Bone quantity was measured by dual-energy X-ray absorptiometry (DXA), which determined the BMD (g/cm^2^) of the lumbar spine (L1 to L4), femoral neck, and hips. Each patient was placed in the center of the table with the spine straight, and a triangular spacer was placed between the lower legs to rotate the hips. The vertebrae were excluded from the analysis if there were severe degenerative sclerotic changes (difference between adjacent vertebral bodies ≥1 standard deviation), compression fractures, or metallic implants. The BMD precision error (coefficient of variation) at our institution is 1% for the lumbar spine, 1.3% for the femoral neck, and 0.8% for hips. The lowest T-score recorded for the spine and hip and were selected from the National Health and Nutrition Examination Survey database for inclusion in the analysis. T-scores were classified according to the World Health Organization system (≥−1.0, normal; −2.5 < T-score < −1.0, osteopenia; and ≤−2.5, osteoporosis [19]).

Bone quality was based on the TBS. TBS is based on two-dimensional texture analysis. It measures gray-level variations in the DXA image as a method to assess bone trabecular microarchitecture. Lower inter-pixel variation indicates good trabecular microarchitecture and denser bone microstructure leading to lower fracture risk. In contrast, higher inter-pixel variation indicates poor trabecular microarchitecture, a more porous bone microstructure, and higher fracture risk [20]. The TBS was extracted from spine DXA files using TBS iNsight^®^ software (version 3.0.0.0; Med-Imaps, Pessac, France). The BMD and TBS are acquired from the same region of interest in DXA images, i.e., from the anteroposterior lumbar spine. According to Schousboe et al., the severity of damaged trabecular bone can be classified according to the TBS, as follows: normal, ≥1.35; partially degraded, 1.20–1.35; and fully degraded, ≤1.20 (“low TBS”) [21].

### 2.4. Fracture Evaluations

#### Imaging Protocol

VF was assessed on a lateral thoracolumbar spine radiograph acquired using standard radiographic system. Standard lateral thoracolumbar spine radiographs at our institution include levels T8−S1. The diagnosis of VF was based on the Genant scoring system, and a grade 1–3 fracture was taken to indicate VF [22]. All thoracolumbar spine radiographs were taken on the same day as the DXA scan. We classified patients with at least one VF in any vertebral level into the “any VF” group; those with more than one VFs in any vertebral level into the “multiple VFs” group.

### 2.5. Muscle Health Evaluation

#### 2.5.1. Imaging Protocols

The amount of lean and bone mass was evaluated using a single fan-beam DXA scanner (Lunar iDXA; GE Medical Systems, Madison, WI, USA). The scanner software automatically selected the scan mode (standard, thin, or thick) depending on patient body size and BMI. Scans were analyzed using enCORE software (version 15; GE Medical Systems, Madison, WI, USA). The positioning of each patient followed the guidelines of the International Society for Clinical Densitometry [23]. The following TBC parameters (in kg) were obtained based on the DXA analysis: total lean mass (TLM), total fat mass (TFM), and total bone mineral content.

#### 2.5.2. Handgrip Strength Assessment

HS (kg) was measured in triplicate in the dominant hand using a single dynamometer (EH101; Camry, Zhongshan, China). The mean value was used in the analysis.

#### 2.5.3. Definition of Sarcopenia

Muscle health was determined according to the presence of sarcopenia. The definition of sarcopenia of the Asian Working Group for Sarcopenia was used, i.e., a low appendicular skeletal muscle index (ASMI) and low HS. The ASMI was calculated by summing the lean mass values of the four extremities and dividing by height (kg/m^2^). The recommended cut-off point for low ASMI is <7.0 kg/m^2^ for men and <5.4 kg/m^2^ for women. Low HS was defined as <26 kg for men and <18 kg for women [24].

### 2.6. Statistical Analysis

Since the incidence of VF differs by sex, the sexes were compared by ANOVA in terms of bone quantity and quality. The independent variables were age, anthropometric measurements, TBC, risk of falls, and muscle health. Post-hoc tests (Tukey’s test for continuous data and chi-square for categorical data) were also applied. To integrate sarcopenia and bone quality and quantity into the predictive model for asymptomatic VF, it was important to determine factors that significantly predicted VF. Participants with VF (any or multiple) were compared to those without in terms of the above parameters, via the t-test and chi-square test for continuous and discrete variables, respectively. The ability of the significant parameters to predict the risk of any and multiple VFs was determined using multivariate logistic regression analysis. Finally, receiver operating characteristic curve analysis was used to determine the optimal combination of factors for predicting VF in each sex. Statistical analysis was performed using R software (version 3.6.2; R Foundation for Statistical Computing, Vienna, Austria) and *p* < 0.05 was taken to indicate statistical significance.

## 3. Results

### 3.1. Demographic Data

A total of 600 participants were included in the analysis; their demographic characteristics are listed in Table 1. There were 186 males and 414 females, with a mean age of 74.30 and 72.83 years, respectively. Despite both sexes having a normal mean BMI (24.00 and 23.90, respectively), the males had a significantly higher TLM, lower TFM, and higher bone mineral content (all *p* < 0.001). However, despite their higher TLM, the males had significantly poorer muscle health than the females; a higher proportion of males had a low ASMI (54.3%, *p* < 0.001) and sarcopenia (36.6%, *p* < 0.001).

There were 257 participants with any VF (87 males) and 136 with multiple VFs (47 males). Although there was no significant difference between the sexes in the rate of any or multiple VFs, a significantly higher proportion of males had sarcopenia in the any and multiple VF groups (49.4 vs. 12.9%, *p* < 0.001, and 48.9 vs. 14.6%, *p* < 0.001, respectively). Notably, males showed significantly better bone quality and quantity than females and were less likely to have a partially degraded TBS or fully degraded TBS, or osteoporosis, despite being more likely to have osteopenia. Finally, there was no significant difference between males and females in the risk of falls.

### 3.2. Associations between Muscle Health and Bone Quantity and Quality

The complete results of the analysis of the associations among TBC, muscle health (sarcopenia), bone quantity (osteoporotic category), and bone quality (TBS) are provided in Table 2.

#### 3.2.1. Male Participants

Male participants with osteoporosis had significantly lower TLM than those in the normal category (*p* < 0.001). A similar association was found between bone quality and TLM (*p* = 0.002). Although a significantly higher proportion of males with osteoporosis had sarcopenia compared to those with osteopenia (47% vs. 22%), there was no significant association between bone quality and sarcopenia (*p* = 0.053). Notably, poor bone quantity also showed a significant correlation with lower TFM (*p* < 0.001).

#### 3.2.2. Female Participants

In the female participants, both poor bone quantity and quality showed a significant association with lower TLM (*p* < 0.001 and *p* = 0.02, respectively). Regarding the relationships of muscle health with bone quantity and quality, there was a borderline significant association between bone quality and sarcopenia (*p* = 0.049), but no significant association between osteoporosis and sarcopenia. Notably, HS was lower in the osteoporotic and fully degraded TBS subgroups compared to the osteopenia and partially degraded TBS subgroups, respectively. Lastly, the osteoporosis and fully degraded TBS female subgroups showed no significant difference in TFM.

### 3.3. Comparison of Participants with and without Asymptomatic Vertebral Fractures

The results of the analysis comparing participants with and without asymptomatic VF (any or multiple VF) are detailed in Table 3.

#### 3.3.1. Male Participants

Although there was no significant difference in bone quantity between participants with and without VF, significantly lower bone quality and TBS was found between the no VF and any VF subgroups, and between the multiple VFs and no multiple VFs subgroups (*p* = 0.032 and *p* = 0.009, respectively). Moreover, the rate of sarcopenia in participants with any VF was higher than that in participants with no VFs (49%, *p* = 0.001), while there was no such significant difference between the multiple VFs and no multiple VFs subgroups. Notably, males with any VF were older than those with no VFs, and males with multiple VFs were older than those without multiple VFs (*p* = 0.034 and *p* = 0.03, respectively). Risk of falls did not vary according to the presence of VF.

#### 3.3.2. Female Participants

The mean TBS was significantly lower in participants with versus without multiple VFs (*p* = 0.047) but not between those with any VF and those with no VFs. Moreover, the proportion of participants with a low HS was significantly higher in the any VF versus no VFs subgroup (*p* = 0.016) and in the multiple VFs versus no multiple VFs subgroups (*p* < 0.001). Sarcopenia incidence did not vary according to the presence of VF. Similar to males, Females with any and multiple VFs were older than those with no VFs and no multiple VFs, respectively (both *p* < 0.001). No significant difference was found in bone quantity between participants with and without VF.

### 3.4. Prediction of Vertebral Fracture Risk

The ability of bone quantity, bone quality and muscle health to predict asymptomatic VF is shown in Table 4. In males, osteoporotic category was not a significant independent predictor of any or multiple VFs. However, both a fully degraded TBS (odds ratio (OR): 11.302, 95% confidence interval (CI): 1.847–219.505, *p* = 0.028) and presence of sarcopenia (OR: 2.820, CI: 1.469–5.533, *p* = 0.002) were significant predictors of any VF. Moreover, a fully degraded TBS (OR: 15.158, CI: 3.462–106.173, *p* = 0.001) and presence of sarcopenia (OR: 1.981, CI: 1.008–3.899, *p* = 0.047) were significant predictors of multiple VFs in males, but not in females.

**Figure 1:**
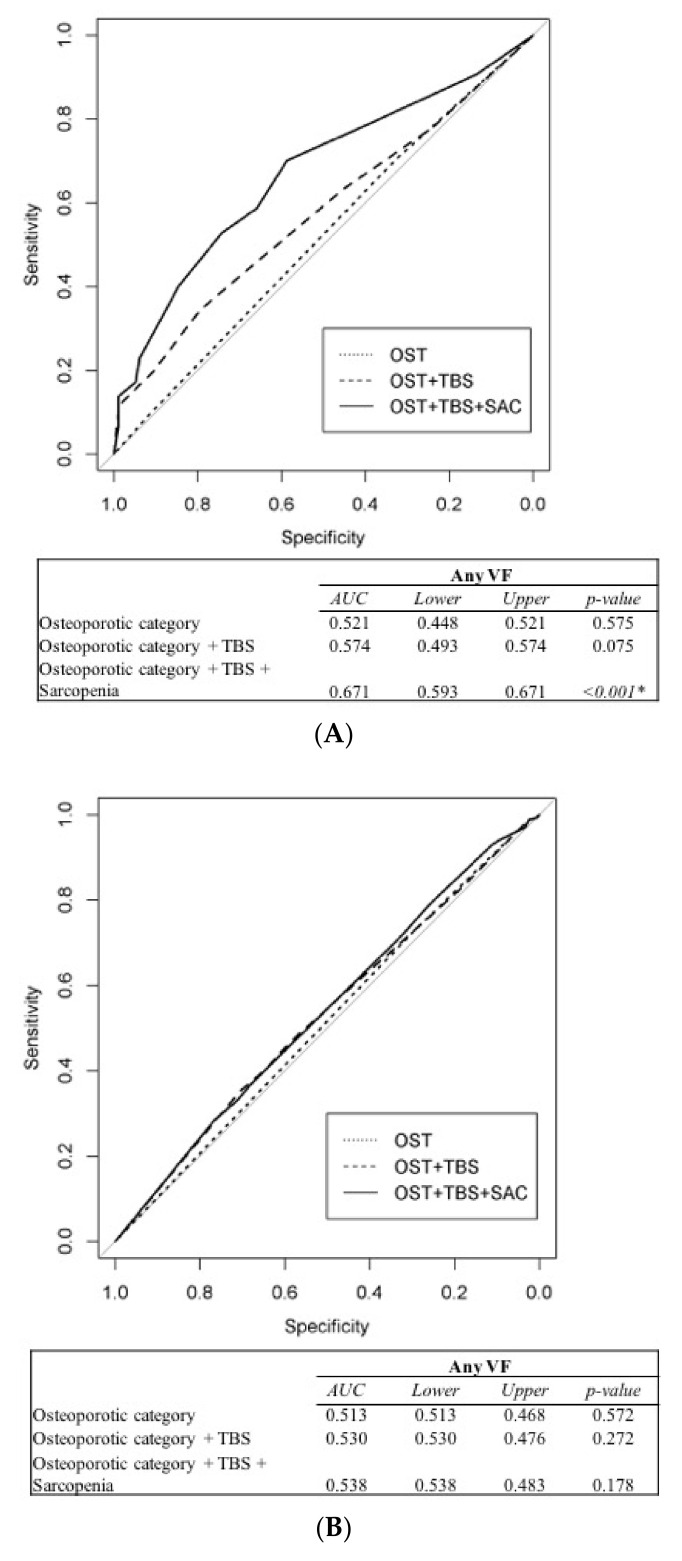

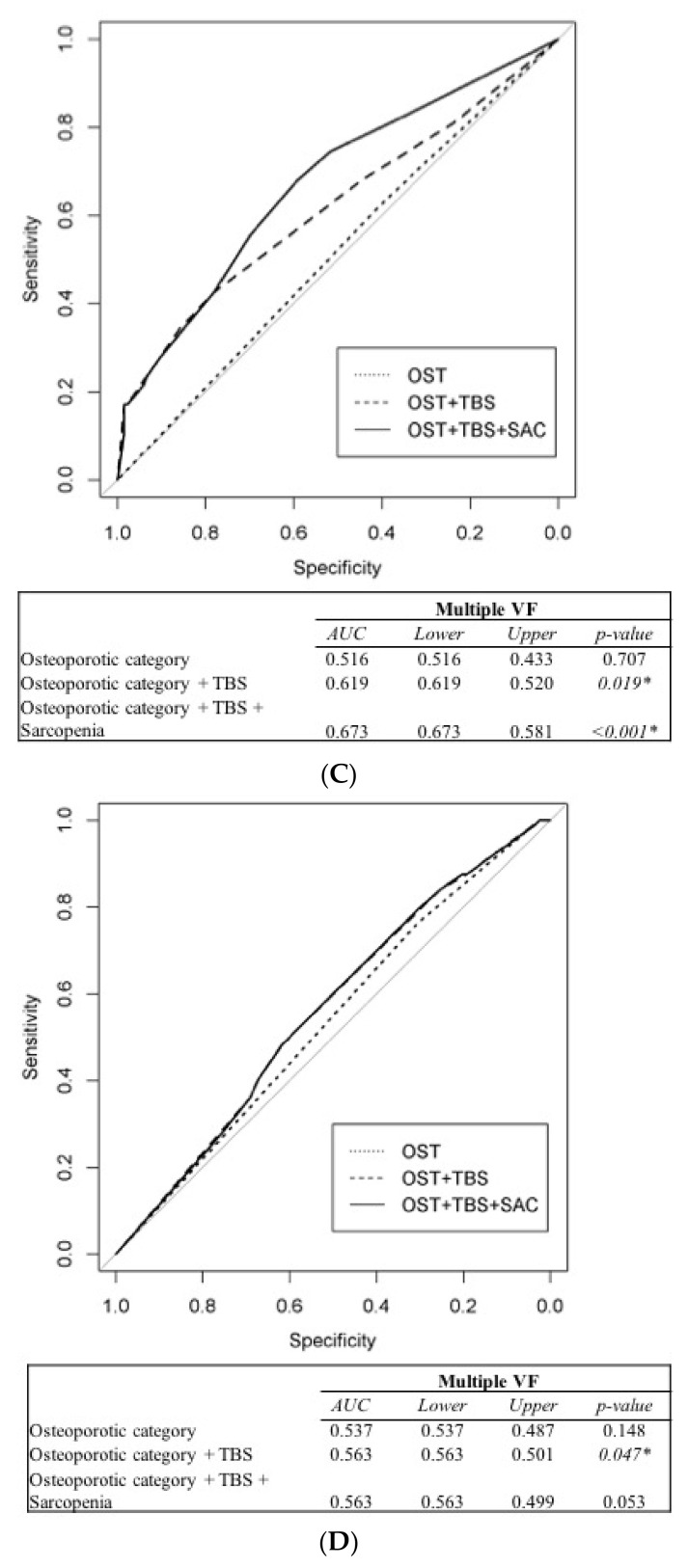
Area under curve (AUC) values for any vertebral fracture (VF) for (**A**) males and (**B**) females, and for multiple VFs for (**C**) males and (**D**) females. OST: osteoporotic category, TBS: trabecular bone score, SAC: sarcopenia. * Significant *p*-value.

The ability of muscle health, and the combination of bone quantity and quality, to predict VF was determined using receiver operating characteristic curve analysis (Figure 1). The results supported the notion that osteoporotic category alone is insufficient for predicting asymptomatic VF. However, in males, osteoporotic category in combination with TBS predicted multiple VFs (area under the curve (AUC): 0.619, *p* = 0.019), although the combination of osteoporosis category, TBS, and sarcopenia status had even better predictive performance in terms of both any (AUC: 0.671, *p* < 0.001) and multiple (AUC: 0.673, *p* < 0.001) VFs. In females, only the combination of osteoporotic category and TBS predicted multiple VFs (AUC: 0.563, *p* = 0.047). Sarcopenia status did not provide any additional predictive power in females.

## 4. Discussion

This study aimed to determine the effects of muscle health on bone quantity and quality and integrate these factors into a predictive model for asymptomatic VF. Even though strong correlations of TLM with bone quantity and quality were seen in both sexes, sarcopenia was only associated with bone quantity in males, and only with bone quality in females (Table 2). When participants with and without VF were compared, significantly lower bone quality and a higher rate of sarcopenia were seen in male participants with any VF. In females, significantly lower bone quality and a higher rate of low HS were seen in participants with multiple VFs (Table 3). Bone quantity showed no significant difference between participants with and without VF, for males or females. Furthermore, when bone quantity, bone quality, and muscle health were combined, bone quantity was a poor predictor of asymptomatic VF in both sexes, while a fully degraded TBS and presence of sarcopenia were significant predictors of both any and multiple VFs in males. Thus, bone quantity alone appears insufficient to predict asymptomatic VF, so it is essential to integrate bone quality and muscle health into models predicting asymptomatic VF in males to allow early diagnosis and prompt treatment, thus lowering the risk of future fracture and improving quality of life. Unfortunately, the above factors had low or negligible predictive power in females.

In this study, TLM was significantly associated with bone quantity and quality in both males and females. Participants with poor bone quantity and quality were more likely to have less TLM; similar results were found in previous studies [25,26]. Locquet et al. reported that a decline in the skeletal muscle mass index was associated with a lower spine BMD (OR = 2.12), hip BMD (2.42), and TBS (3.99) [25]. One possible reason why the muscle and bone indices showed close associations might be the mechanic interactions that exist between these two organs, where muscle tissue shows static and dynamic loading on bone [27,28]. Static loading, due to the weight of the muscle, is caused by gravitational force. When there is a larger amount of muscle, mechanical stress on the bone is higher [27]. Dynamic loading, meanwhile, is due to muscle contraction (e.g., HS), with tendons exerting tension on the bone [28,29]. Tension applied to bone is sensed by osteocytes, which then differentiate into osteoblasts to improve bone quantity and quality [27]. In this study, the incidence of sarcopenia was found to be significantly different between the males with osteopenia and osteoporosis, and between the females with partially degraded and fully degraded TBS. Above, it was suggested that the impact of muscle health on bone quantity and quality differed according to sexes, and sarcopenia was associated only with bone quantity in males and only with bone quality in females. It is interesting that low HS was associated with both osteoporosis and a fully degraded TBS in females (Table 2). Loss of HS may be associated with the loss of bone quantity and quality, as reported by a previous study, with women being more prone to decreasing HS than decreasing muscle mass with age [30]. The loss of HS can be evaluated clinically without the need for additional radiographic images, which suggests a potential role in the clinical prediction of asymptomatic VF in older adult women with sarcopenia. However, loss of HS could be caused by factors other than sarcopenia, such as neurogenic disorders; thus, HS alone may be insufficient as an index of overall muscle health.

The available literature on the utility of sarcopenia for predicting asymptomatic VF is limited, while previous research concerning sarcopenia and symptomatic VF is equivocal. One study showed that the prevalence of sarcopenia was higher in patients with VF [31] and Hida et al. [32] reported that sarcopenia was an independent risk factor for acute VF (OR: 1.96). However, Trajanoska et al. [33] found that sarcopenia did not increase the risk of VF. Here, we demonstrated that sarcopenia was a significant predictor of both any (OR: 2.820) and multiple (OR: 1.981) asymptomatic VFs, but only in male participants. However, we believe that our study still supports a causative role of sarcopenia in VF. Many previous reports indicated that sarcopenia might be the result of symptomatic VF. For instance, patients with symptomatic VF may develop anorexia and require prolonged bed rest due to back pain and spinal deformity, where a combination of malnutrition and immobility may eventually lead to sarcopenia [17,34]. Differing to the above studies, our study included only asymptomatic participants, so any influence of VF-related symptoms was ruled out. Nevertheless, sarcopenia remained a significant predictor of VF risk. Therefore, our results support an essential role of sarcopenia in VF, where poor muscle health is associated with less spine support. Ignasiak et al. [35] reported an increase in the compressive force exerted on the vertebra (by up to 36%, or 318 N) at the level of the upper thoracic spine, and an increase in shear loading (by up to 75%, or 176 N) along the whole spine. This substantial compression and shear loading of the spine eventually leads to VF in sarcopenia patients. Furthermore, most studies established the presence of VF based on medical record review, which may result in underdiagnosis of VFs. In contrast, all participants in our study underwent standard spine radiography for diagnosing VF accurately. Notably, although the female participants with VF were more likely to have a low HS (Table 3), sarcopenia had only a limited ability to predict VF in females. Harris et al. [36] obtained a similar result; they found that sarcopenia was not a risk factor for VF in women with osteoporosis. This sex difference cannot be explained based on the currently available data, and thus merits additional study.

Furthermore, this study was the first to integrate both bone quality and sarcopenia into a model for predicting asymptomatic VF. We demonstrated that osteoporosis alone was insufficient to predict VF in both sexes, such that integration of TBS into the model is essential. Integration of sarcopenia status further enhanced the accuracy of the model, although only for the male participants. Thus, if a male patient has a fully degraded TBS and sarcopenia, clinicians should suspect asymptomatic VF and thus perform additional workup. Notably, although sarcopenia was not an independent predictor of asymptomatic VF in females, integration of TBS may enhance the predictive accuracy of models for multiple VFs (Figure 1D).

Some limitations of this study should be mentioned. First, walking speed was not measured. Dysmotility syndrome has been reported to elevate VF risk, so low gait speed may also be associated with VF [37]. Second, the proportion of male participants with a fully degraded TBS was relatively small compared to that of the female participants. Third, there were few cases of sarcopenia within the normal population. Fourth, a patient with a history of alcohol, tobacco, drug dosage, comorbidities, physical activity level, and a previous fracture was not recorded and included in the analysis, which may lead to selection bias. Finally, this study included only Asians, so studies in Western societies should also be performed.

## 5. Conclusions

Significant associations were observed between TLM and bone quantity and quality. Despite sex differences in the associations of sarcopenia with bone quantity and quality, sarcopenia should be included in predictive models for VF. A fully degraded TBS and sarcopenia were the only significant predictors of asymptomatic VF in males, in whom VF risk assessment may facilitate early diagnosis.

## Figures and Tables

**Table 1:** Demographic data of the 600 participants.

	Male (*n* = 186)				Female (*n* = 414)				
	Num. of Patients	%	Mean	SD	Num. of Patients	%	Mean	SD	*p*-Value
Age (years)			74.30	7.14			72.82	7.37	0.02
Weight (kg)			63.00	10.26			55.20	8.89	<0.001 *
Height (cm)			161.92	6.59			151.90	5.83	<0.001 *
BMI (kg/m^2^)			24.00	3.44			23.90	3.50	0.75
TFM (kg)			18.33	5.69			21.07	9.72	<0.001 *
TLM (kg)			41.85	5.89			32.12	4.75	<0.001 *
TBMC (kg)			2.30	0.39			1.66	0.26	<0.001 *
ASMI (kg/m^2^)			6.93	0.98			5.88	0.79	<0.001 *
Risk of fall	9	4.8			37	9.0			0.11
Vertebral fractures									
Any VF	87	47.0			170	41.4			0.23
w sarcopenia	43	49.4			22	12.9			<0.001 *
w/o sarcopenia	44	50.6			148	87.1			
Multiple VFs	47	25.4			89	21.7			0.37
w sarcopenia	23	48.9			13	14.6			<0.001 *
w/o sarcopenia	24	51.1			76	85.4			
Bone quantity (osteoporotic category)									<0.001 *
Normal	16	8.6			8	1.9			
Osteopenia	60	32.3			111	27.0			
Osteoporosis	110	59.1			292	71.0			
Bone quality (trabecular bone score)									<0.001 *
Normal	92	49.7			39	9.5			
Partially degraded	83	44.9			217	52.8			
Fully Degraded	10	5.4			155	37.7			
Muscle health									
Low ASMI	101	54.3			117	28.3			<0.001 *
Low HS	105	56.5			197	47.9			0.07
Sarcopenia	68	36.6			58	14.1			<0.001 *

* Significant *p*-value. SD: standard deviation, BMI: body mass index, TFM: total fat mass, TLM: total lean mass, TBMC: total bone mineral content, ASMI: appendicular skeletal muscle index, w: with, w/o: without, HS: handgrip strength, VF: vertebral compression fracture. Low ASMI is <7.0 kg/m^2^ for men and <5.4 kg/m^2^ for women; low HS is <26 kg for men and <18 kg for women. Sarcopenia is defined by a low ASMI and HS. Osteoporotic category: T-score ≥ −1.0 is normal, −2.5 < T-score < −1.0 denotes osteopenia, and T-score ≤ −2.5 denotes osteoporosis. Trabecular bone score: ≥1.35 is normal, 1.2–1.35 denotes partially degraded trabecular bone, and ≤1.2 denotes fully degraded trabecular bone.

**Table 2:** The associations of body composition with bone quality and quantity.

Osteoporotic Category	Normal		Osteopenia		Osteoporosis		*p*-Value
Males	Mean		Mean		Mean		
Age (years), SD	74.56	6.60	74.45	6.84	74.18	7.44	0.962
Weight (kg), SD	73.63 ^	11.04	66.59 ^^	9.06	59.49 ^^^	9.03	<0.001 *
Height (cm), SD	163.85	5.94	163.61 ^^	6.25	160.72	6.64	0.01 *
BMI (kg/m^2^), SD	27.41 ^	3.67	24.86 ^^	2.99	23.04 ^^^	3.22	<0.001 *
TFM (kg), SD	23.33 ^	5.64	19.52 ^^	4.97	16.95 ^^^	5.55	<0.001 *
TLM (kg), SD	46.95	6.64	43.93 ^^	5.37	39.97 ^^^	5.24	<0.001 *
Risk of fall (*n*), %	2 ^	13%	0	0%	7	6%	0.022 *
Muscle health							
Low ASMI (*n*), %	4	25%	27	45% ^^	70	64% ^^^	0.003 *
Low HS (*n*), %	9	56%	27	45%	69	63%	0.085
Sarcopenia (*n*), %	3	19%	13	22% ^^	52	47%	0.001 *
Females	Mean		Mean		Mean		
Age (years), SD	69.50	6.91	71.25 ^^	6.10	73.50	7.72	0.01 *
Weight (kg), SD	62.73 ^	5.98	58.57 ^^	9.26	53.72 ^^^	8.36	<0.001 *
Height (cm), SD	157.31 ^	6.66	153.42 ^^	5.51	151.17 ^^^	5.76	<0.001 *
BMI (kg/m^2^), SD	25.38	2.42	24.9 ^^	3.81	23.49	3.32	0.001 *
TFM (kg), SD	23.35	3.84	22.48	6.20	20.47	10.82	0.145
TLM (kg), SD	36.23	4.81	33.39 ^^	4.86	31.53 ^^^	4.57	<0.001 *
Risk of fall (*n*), %	0	0%	8	7%	29	10%	0.628
Muscle health							
Low ASMI (*n*), %	2	25%	29	26%	86	29%	0.859
Low HS (*n*), %	5	63%	38	34% ^^	154	53%	0.002 *
Sarcopenia (*n*), %	0	0%	11	10%	47	16%	0.209
**Trabecular Bone Score**	**Normal**		**Partially Degraded**		**Fully Degraded**		***p*-Value**
Males	Mean		Mean		Mean		
Age (years), SD	73.80	7.28	74.86	7.08	75.20	6.36	0.580
Weight (kg), SD	65.05 ^	10.75	61.33	9.48	57.27	8.20	0.011 *
Height (cm), SD	162.36	6.02	161.70	7.06	158.45	6.34	0.193
BMI (kg/m^2^), SD	24.64	3.62	23.45	3.20	22.82	3.01	0.039 *
TFM (kg), SD	18.46	5.78	18.42	5.67	15.96	4.98	0.409
TLM (kg), SD	43.29 ^	6.02	40.72	5.39	38.08 ^^^	5.89	0.002 *
Risk of fall (*n*), %	4	4%	5	6%	0	0%	0.843
Muscle health							
Low ASMI (*n*), %	44	48%	48	58%	8	80%	0.113
Low HS (*n*), %	50	54%	47	57%	8	80%	0.334
Sarcopenia (*n*), %	29	32%	32	39%	7	70%	0.053
Females	Mean		Mean		Mean		
Age (years), SD	70.36	5.67	72.56	7.49	73.80 ^^^	7.44	0.025 *
Weight (kg), SD	59.85 ^	8.70	55.53	8.35	53.58 ^^^	9.25	<0.001 *
Height (cm), SD	152.97	7.36	152.04	5.67	151.43	5.62	0.297
BMI (kg/m^2^), SD	25.60 ^	3.48	24.01	3.35	23.33 ^^^	3.59	0.001 *
TFM (kg), SD	22.73	5.49	21.67	11.88	19.80	6.58	0.099
TLM (kg), SD	33.11	6.30	32.53 ^^	4.08	31.31	5.08	0.02 *
Risk of fall (*n*), %	3	8%	15	7%	19	12%	0.203
Muscle health							
Low ASMI (*n*), %	7	18%	57	26%	53	34%	0.077
Low HS (*n*), %	16	41%	93	43% ^^	88	57%	0.020 *
Sarcopenia (*n*), %	3	8%	25	12% ^^	30	19%	0.049 *

* Significant *p*-value. The post-hoc test results can be described as follows (where *p* < 0.05 was taken to indicate significance): ^ significant difference between osteopenia and normal; ^^ significant difference between osteopenia and osteoporosis; and ^^^ significant difference between osteoporosis and normal. SD: standard deviation, BMI: body mass index, TFM: total fat mass, TLM: total lean mass, TBMC: total bone mineral content, ASMI: appendicular skeletal muscle index, HS: handgrip strength. Low ASMI is <7.0 kg/m^2^ for men and <5.4 kg/m^2^ for women; low HS is <26 kg for men and <18 kg for women. Sarcopenia is defined by a low ASMI and HS. Osteoporotic category: T-score ≥ −1.0 is normal, −2.5 < T-score < −1.0 denotes osteopenia, and T-score ≤ −2.5 denotes osteoporosis. Trabecular bone score: ≥1.35 is normal, 1.2–1.35 denotes partially degraded trabecular bone, and ≤1.2 denotes fully degraded trabecular bone.

**Table 3:** Comparison between participants with and without vertebral compression fractures (VFs).

	Any VF		No VF		*p*-Value	Multiple VFs		No Multiple VFs		*p*-Value
Males										
Age (years), SD	75.47	7.99	73.23	6.19	0.034 *	76.23	8.37	73.62	6.60	0.03 *
Weight (kg), SD	62.07	9.82	63.73	10.65	0.271	62.02	10.00	63.27	10.38	0.467
Height (cm), SD	161.30	6.34	162.39	6.78	0.260	160.39	6.91	162.38	6.41	0.086
BMI (kg/m^2^), SD	23.84	3.23	24.14	3.65	0.547	24.08	3.28	23.97	3.52	0.839
TFM (kg), SD	18,227.28	5787.95	18,409.71	5651.66	0.829	18,399.63	5727.10	18,298.13	5713.17	0.917
TLM (kg), SD	41,008.78	5745.54	42,528.63	5950.84	0.079	40,795.42	6072.50	42,160.76	5806.37	0.182
Risk of fall (*n*), %	7	8%	2	2%	0.086	3	6%	6	4%	0.695
Bone quantity										
Bone mineral density (g/cm^2^)	0.77	0.11	0.77	0.13	0.882	0.79	0.11	0.76	0.12	0.372
Bone quality										
Trabecular bone score	1.33	0.10	1.36	0.08	0.032 *	1.32	0.11	1.36	0.08	0.009 *
Muscle health										
Low ASMI (*n*), %	57	66%	44	45%	0.008 *	30	64%	71	51%	0.193
Low HS (*n*), %	62	71%	42	43%	<0.001 *	31	66%	73	53%	0.165
Sarcopenia (*n*), %	43	49%	25	26%	0.001 *	23	49%	45	33%	0.067
Females										
Age (years), SD	74.82	7.72	71.41	6.78	<0.001 *	76.88	7.99	71.70	6.78	<0.001 *
Weight (kg), SD	55.29	8.67	55.14	9.05	0.863	54.35	8.09	55.44	9.09	0.278
Height (cm), SD	151.60	5.83	152.11	5.84	0.388	150.73	6.04	152.22	5.74	0.039 *
BMI (kg/m^2^), SD	24.03	3.35	23.82	3.61	0.546	23.92	3.24	23.90	3.58	0.967
TFM (kg), SD	21,619.22	13,324.25	20,681.37	6003.69	0.336	19,894.98	6084.48	21,393.87	10,490.67	0.198
TLM (kg), SD	32,111.69	5307.66	32,134.30	4330.71	0.962	31,929.38	4850.85	32,179.00	4731.80	0.666
Risk of fall (*n*), %	19	11%	18	7%	0.263	11	12%	26	8%	0.298
Bone quantity										
Bone mineral density (g/cm^2^)	0.66	0.13	0.68	0.11	0.144	0.66	0.11	0.67	0.12	0.649
Bone quality										
Trabecular bone score	1.22	0.09	1.23	0.08	0.340	1.21	0.09	1.23	0.09	0.047 *
Muscle health										
Low ASMI (*n*), %	43	25%	74	31%	0.277	21	24%	96	30%	0.880
Low HS (*n*), %	94	55%	103	43%	0.016 *	58	65%	139	43%	<0.001 *
Sarcopenia (*n*), %	22	13%	36	15%	0.668	13	15%	45	14%	1.000

* Significant *p*-value. SD: standard deviation, BMI: body mass index, TFM: total fat mass, TLM: total lean mass, TBMC: total bone mineral content, ASMI: appendicular skeletal muscle index, HS: handgrip strength. Low ASMI is <7.0 kg/m^2^ for men and <5.4 kg/m^2^ for women; low HS is <26 kg for men and <18 kg for women. Sarcopenia is defined by a low ASMI and HS.

**Table 4:** Predictive power of different parameters for vertebral compression fracture (VF).

	Males				Females			
	OR	Lower	Upper	*p*-Value	OR	Lower	Upper	*p*-Value
**Any VF**								
Osteoporotic category								
Normal	1.000				1.000			
Osteopenia	0.712	0.220	2.292	0.565	2.058	0.426	15.019	0.404
Osteoporosis	0.517	0.159	1.651	0.263	2.141	0.439	15.736	0.382
Trabecular bone score								
Normal	1.000				1.000			
Partially degraded	1.264	0.661	2.433	0.480	0.947	0.450	2.035	0.887
Fully Degraded	11.302	1.847	219.505	0.028 *	1.164	0.531	2.597	0.707
Sarcopenia								
w	1.000				1.000			
w/o	2.820	1.469	5.533	0.002 *	0.805	0.446	1.423	0.461
**Multiple VFs**								
Osteoporotic category								
Normal	1.000				-	-	-	-
Osteopenia	0.933	0.273	3.748	0.916	1.000			
Osteoporosis	1.074	0.343	4.079	0.908	1.301	0.763	2.290	0.346
Trabecular bone score								
Normal	1.000				1.000			
Partially degraded	1.203	0.588	2.468	0.611	1.130	0.490	2.941	0.787
Degraded	15.158	3.462	106.173	0.001 *	1.537	0.659	4.039	0.346
Sarcopenia								
w	1.000				1.000			
w/o	1.981	1.008	3.899	0.047 *	1.053	0.522	2.002	0.880

* Significant *p*-value. OR: odds ratio, BMI: body mass index, TFM: total fat mass, TLM: total lean mass, TBMC: total bone mineral content, ASMI: appendicular skeletal muscle index. Sarcopenia is defined by a low ASMI and HS. Osteoporotic category: T-score ≥ −1.0 is normal, −2.5 < T-score < −1.0 denotes osteopenia, and T-score ≤ −2.5 denotes osteoporosis. Trabecular bone score: ≥1.35 is normal, 1.2–1.35 denotes partially degraded trabecular bone, and ≤1.2 denotes fully degraded trabecular bone.

## Data Availability

The data presented in this study are available on request from the corresponding author.
